# Modeling and Research on Power System of Distributed Sensor Networks for Long Streamers in Marine Seismic Exploration

**DOI:** 10.3390/s20010028

**Published:** 2019-12-19

**Authors:** Hongwei Yu, Kezhu Song, Junfeng Yang, Chuan Wu, Ke Zhong, Wengui Lv

**Affiliations:** 1 Department of Modern Physics, University of Science and Technology of China, Hefei 230026, China; yhw1993@mail.ustc.edu.cn (H.Y.); yangjf@ustc.edu.cn (J.Y.); callmewu@mail.ustc.edu.cn (C.W.); ftall@mail.ustc.edu.cn (K.Z.); lwgui@mail.ustc.edu.cn (W.L.); 2 State Key Laboratory of Particle Detection and Electronics, University of Science and Technology of China, Hefei 230026, China

**Keywords:** marine seismic exploration, power supply network, long streamer, large-scale sensor networks

## Abstract

The electric power system plays an important role in sensor networks. In the marine seismic exploration streamer system (MSESS), an underwater power system transmits high-voltage direct current to all nodes in the streamer through a daisy chain structure. As offshore oil exploration develops toward deep water, it is necessary to study long streamers with large-scale sensor networks for deep water exploration. When the length of a streamer is increased to a certain value, the output current of the power supply increases sharply. This results in the activation of the overcurrent protection and the power supply shuts down. This paper puts forward an accurate model for an underwater power system applied to MSESS. Using the Newton iteration algorithm and a reverse algorithm, equations established by the model are solved and laboratory test results are used to verify the accuracy of the model. Based on simulation and analysis of the model, we explain why the power system crashes when the streamer is too long. Software that can quickly calculate the maximum number of nodes (the maximum length with which the system works normally) is developed and it is significant for the design of MSESS. The method of research could also be applied to relevant work such as large-scale sensor networks with daisy-chaining power supply in land seismic exploration.

## 1. Introduction

In recent years, new oil and gas resources have come mainly from the sea, particularly from deep water and ultra-deep water areas [1,2,3,4]. As marine oil and gas exploration gradually develops into deep water areas, the exploration targets are gradually moving from the ocean surface to medium depths, which puts more stringent requirements on seismic exploration technology [5,6,7].

**Figure 1:**
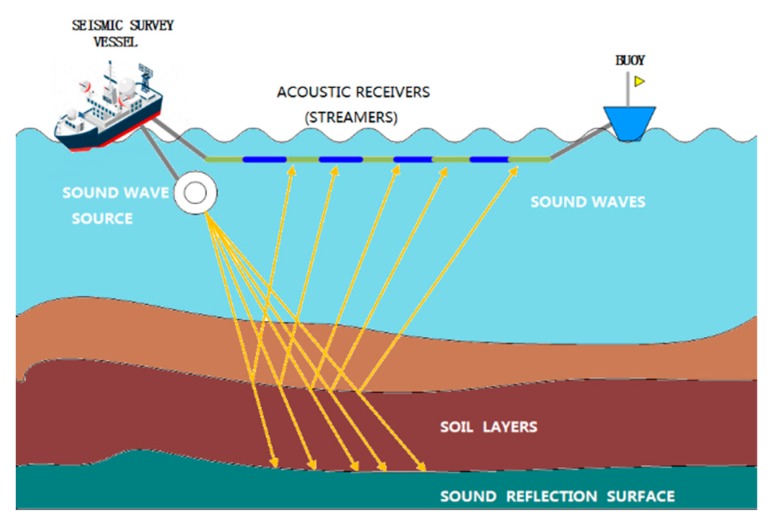
Working process of marine seismic exploration.

The basic method of marine seismic exploration is to create artificial seismic waves through air guns in seawater. Sensor networks in the streamer collect seismic waves reflected from the submarine strata. After being processed, the original record information is used to infer the underground structure of the subsea strata [8,9,10,11,12]. The working process is shown in Figure 1. For the effective exploration and development of oil and gas in deep water environments, it is necessary to study long streamers. The increase in streamer length makes it difficult to provide the data acquisition station (DAS) with an effective power supply.

Large-scale sensor networks and poor working conditions at sea necessitate a system with a convenient and stable power supply [13,14,15]. Most of the exploration instruments on the market adopt a daisy chain structure to transfer high voltage to single nodes. Direct current (DC)-to-DC converters transform high voltage to low voltage, which powers the acquisition station [16,17]. In actual operation, these kinds of power system normally work in mesoscale sensor networks. As the quantity of data acquisition nodes increases, power consumption appears to increase in a non-linear fashion, and a rapid increase in current causes the system crash. For instance, the maximum length of the streamer developed by the China National Offshore Oil Corp (CNOOC) named “HQI-Seis” can reach only 7 km with a tracking spacing of 3.125 m [18,19,20]. When one more node is added, the output current of the power supply on the geophysical survey vessel increases sharply. Once this happens, the overcurrent protection function of the power supply engages and the power supply disconnects the output. The inability of “HQI-Seis” to support a longer length has confused designers.

In order to study long streamers and analyze the inability of the system to support infinite nodes, we design a power dissipation model with line loss for marine seismic exploration sensor networks. Non-linear equations established by the model are solved by Newton iteration algorithm and reverse algorithm. Simulation and testing verify the accuracy of the model. Based on the study of the model, the reason why the marine seismic exploration streamer system (MSESS) can only support a finite number of nodes is found. To provide guidance for the design of the power system, software is developed to predict the maximum number of nodes under specific conditions.

## 2. Structure of Marine Seismic Exploration Streamer (MSESS)

**Figure 2:**
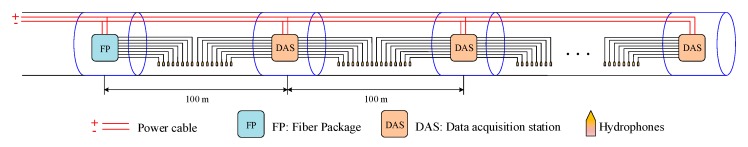
Internal power supply structure of streamer.

Marine seismic exploration sensor networks are composed of several towlines. The internal power supply structure of one single towing data acquisition system is presented in Figure 2. There are multiple DASs in the streamer. Every two DASs is 100 m apart and DASs are responsible for collecting signals from a total of 16 sensors. The sensors include piezoelectric ceramic sensors and microelectro mechanical systems (MEMS) sensors, which receive seismic wave signals reflected by the strata.

**Figure 3:**
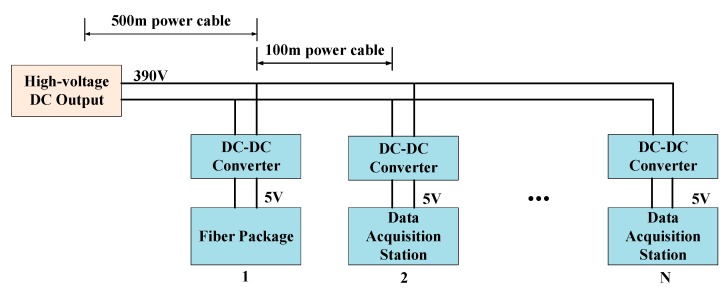
Architecture of the power system.

The power system of MSESS is high-voltage direct current output in a daisy chain distribution pattern. A low-resistance power cable with nodes spaced 100 m apart is used to transfer electricity. The node is responsible for data acquisition and transmission. The first node (fiber package) is located 500 m from the power supply on the geophysical survey vessel, which utilizes optical fiber to transmit data. DASs use twisted-pair cables to transmit data. On each node, a DC-to-DC converter is used to transform high voltage on the power cable to usable voltage for the DASs. Figure 3 illustrates the structure of the power system.

## 3. Power Supply Model

In this section, we set up a model to analyze voltage distribution and current distribution on the power cables. According to the model, non-linear equations are established and two algorithms to solve the equations are proposed. We give a detailed description about how to set up the model in Section 3.1. The algorithms are detailed in Section 3.2.

### 3.1. Setting Up the Model

**Figure 4:**
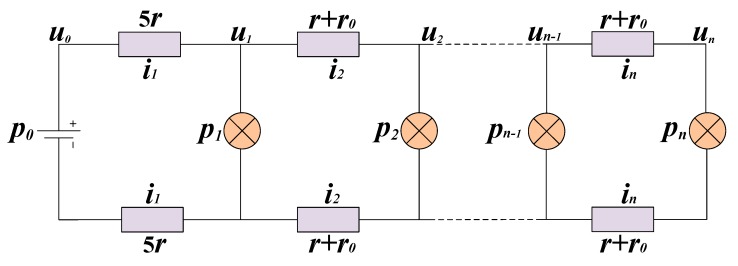
Equivalent model of power system.

According to the architecture of the power system, we create a model to analyze system power consumption and voltage drop on the power cables. In Figure 4, (1, 2, …, *n)* represent the number of nodes. The variable u_0_ is the high-voltage direct current output (HVO) and u_1_~u_n_ are the voltage on the power cable at the terminal of each node. The variable r is the resistance of the 100 m power cable and r_0_ is the turn-on resistance of the metal-oxide-semiconductor field effect transistor (MOSFET) switch, which enables the power supply of the next node. The MOSFET switch also exists in the return path. The variables i_1_~i_n_ are the current on each 100 m power cable. The variable p_0_ stands for the power dissipation of the whole system and p_1_~p_n_ represent the power consumption of each node, including the DC-to-DC converter and the DAS. The variable p_1_ is the power consumption of the fiber package, which is different from DAS. 

On the basis of the Kirchhoff voltage law, Equation (1) is acquired:

(1) $$u_{n-1}=u_{n}+\left(r+r_{0}\right)*i_{n}+\left(r+r_{0}\right)*i_{n}=u_{n}+2*\left(r+r_{0}\right)*i_{n}=u_{n}+\left(R+R_{0}\right)*i_{n}$$

**Figure 5:**
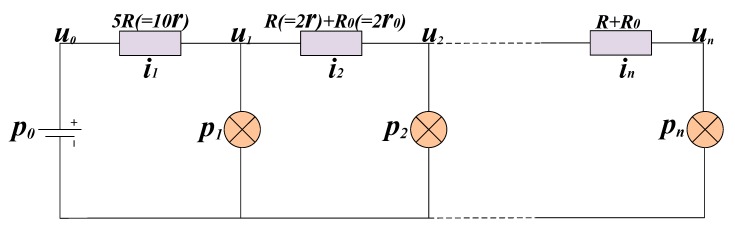
Simplified model of power system.

The model can be simplified as shown in Figure 5. R represents the impedance of a loop of two 100 m power cables while *R*_0_ is the trigger resistance of the loop switch. We used this model to calculate the power consumption (*p*_0_) and voltage drop on the power cable (*u*_1_~*u*_n_). 

Using the Kirchhoff voltage law and the Kirchhoff current law, we can derive Equations (2) and (3). Equations (4) can be derived by combining Equations (2) and (3). The variable *p*_0_ is analyzed as described in Equation (5). In Equation (5), *p*_0_ represents the power dissipation of the whole system and it consists of two parts. One is line loss on power cable, which corresponds to the first part of Equation (5) ($\frac{(u_{0}-u_{1}{)}^{2}}{5R}+\frac{(u_{1}-u_{2}{)}^{2}}{R+R_{0}}+\ldots +\frac{(u_{n-1}-u_{n}{)}^{2}}{R+R_{0}}$). The other is power consumption of DASs, which corresponds to the last part of Equation (5) ($\overset{n}{\underset{i=1}{\sum }}p_{i}$). The next section will discuss methods for solving these equations.

(2) $$\{\begin{array}{c} u_{0}=5i_{1}R+u_{1}\\ u_{1}=i_{2}\left(R+R_{0}\right)+u_{2}\\ \ldots \\ u_{n-1}=i_{n\;}\left(R+R_{0}\right)+u_{n} \end{array}$$

(3) $$\{\begin{array}{c} i_{1}=i_{2}+\frac{p_{1}}{u_{1}}\\ i_{2}=i_{3}+\frac{p_{2}}{u_{2}}\\ \ldots \\ i_{n-1}=i_{n}+\frac{p_{n-1}}{u_{n-1}}\\ i_{n}=\frac{p_{n}}{u_{n}} \end{array}$$

(4) $$\{\begin{array}{l} \frac{p_{1}}{u_{1}}+\frac{u_{1}-u_{2}}{R+R_{0}}=\frac{u_{0}-u_{1}}{5R}\\ \frac{p_{2}}{u_{2}}+\frac{u_{2}-u_{3}}{R+R_{0}}=\frac{u_{1}-u_{2}}{R+R_{0}}\\ \ldots \\ \frac{p_{n-1}}{u_{n-1}}+\frac{u_{n-1}-u_{n}}{R+R_{0}}\frac{u_{n-2}-u_{n-1}}{R+R_{0}}\\ \frac{p_{n}}{u_{n}}=\frac{u_{n-1}-u_{n}}{R+R_{0}} \end{array}$$

(5) $$p_{0}=\frac{(u_{0}-u_{1}{)}^{2}}{5R}+\frac{(u_{1}-u_{2}{)}^{2}}{R+R_{0}}+\ldots +\frac{(u_{n-1}-u_{n}{)}^{2}}{R+R_{0}}+\overset{n}{\underset{i=1}{\sum }}p_{i}$$

### 3.2. Method to Solve Equation

Since the efficiency of the DC–DC converter varies as input voltage changes and the power consumption of DAS remains at a constant value, single-node power consumption *p*_n_ is a function of input voltage *u*_n_, which is expressed as *p*_n_ = *f* (*u*_n_). We tested a DC-to-DC converter in the laboratory and fitted an efficiency curve to the data. The relationship of eff (*n*) and *u*_n_ is shown in Equation (6).

(6) $$eff\left(n\right)=\left(-0.03395u_{n}+91.75\right)/100$$

According to measured values, the power consumption of the fiber package (PCFP) is 2.5 W and the power consumption of DAS (PDAS) is 2.3 W. Using these values, we derived Equations (7) and (8).

(7) $$p_{1}=2.5/eff\left(1\right)$$

(8) $$p_{n}=2.3/eff\left(n\right)$$

Equations (9) are derived by incorporating laboratory data (*u*_0_ = 390 V, R = 0.9 Ω, and *R*_0_ = 0.25 Ω) into Equation (4).

(9) $$\{\begin{array}{c} \frac{2.5}{u_{1}*eff\left(1\right)}\;+\frac{u_{1}-u_{2}}{1.15}=\frac{390-u_{1}}{4.5}\\ \frac{2.3}{u_{2}*eff\left(2\right)}+\frac{u_{2}-u_{3}}{1.15}=\frac{u_{1}-u_{2}}{1.15}\\ \ldots \\ \frac{2.3}{u_{n-1}*eff\left(n-1\right)}+\frac{u_{n-1}-u_{n}}{1.15}=\frac{u_{n-2}-u_{n-1}}{1.15}\\ \frac{2.3}{u_{n}*eff\left(n\right)}=\frac{u_{n-1}-u_{n}}{1.15} \end{array}$$

**Figure 6:**
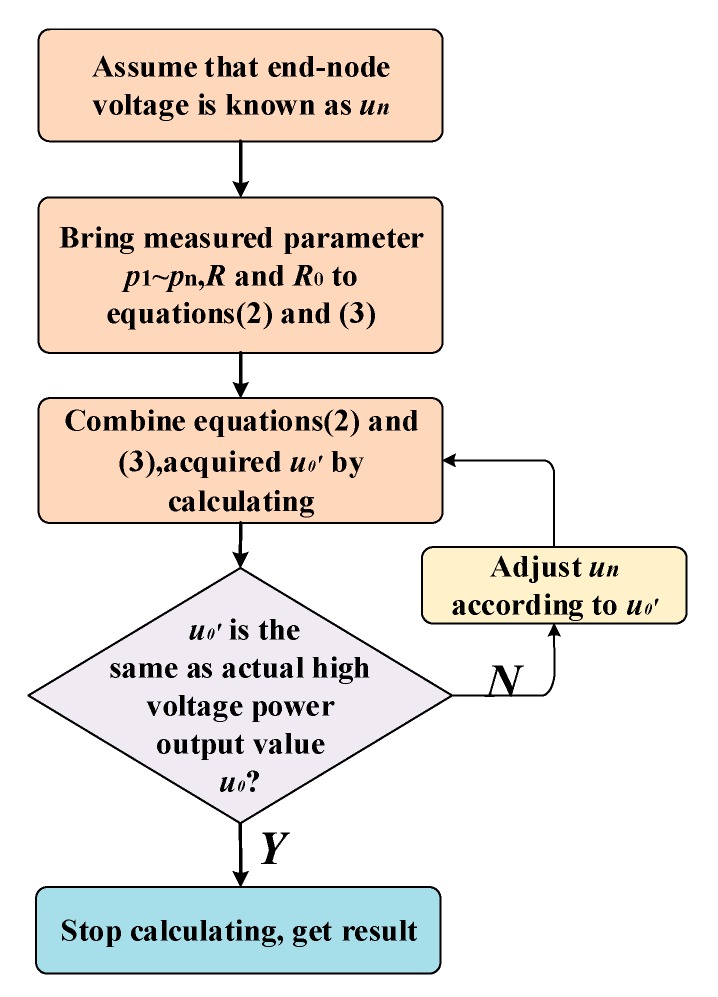
Calculation process of the reverse algorithm.

Equations (9) are a typical system of non-linear equations. Traditional algorithms such as the Newton iteration algorithm are used to solve the system of non-linear equations [21,22,23]. However, some problems still exist. The convergence and performance characteristics of the algorithm are highly sensitive to the initial values and the computational efficiency is low [24,25]. By analyzing Equations (2) and (3), we put forward a new algorithm named “reverse algorithm”. The advantage of the reverse algorithm is that highly accurate results can be calculated quickly. Figure 6 illustrates the solution flow chart.

The pseudocode of reverse algorithm is shown in Algorithm 1. We use a reverse algorithm to obtain *u_1_*~*u_n_* and *i*_1_~*i_n_* in Equations (9). Equations (9) are acquired by Equations (2) and (3). The first step is to choose a suitable value of *u_n_*. In step 2, *i_n_* is acquired by utilizing the last equation in (3). In step 3 to 6, *u_1_*~*u_n__−1_* and *i*_1_~*i_n__−1_* is calculated by Equations (2) and (3). In step 7, we obtain *u_0′_* and compare it with *u*_0_. If the value of *u_0′_* is the same as *u*_0_, calculating process is terminated. Otherwise *u_n_* is adjusted according to *u_0′_* and step 1 to step 7 are repeated till we get the right results.
**Algorithm 1.** Reverse algorithm ()Input (*n*)Output (*u*_1_~*u_n_*, *i*_1_~*i_n_*)Known parameters: *u*_0_, *p*_1_~*p*_n_, *R*, *R*_0_
**do** assume *u_n_* = constant (*u_n_* needs to adjust according to *u*_0_*_′_*)j = *n*, *i_j_* = *p_j_*/*u_j_*,**for** j = *n* to 2*u*_*j*−1_ = i_j_*(*R* + *R*_0_) + *u_j_**i*_*j*−1_ = *i_j_* + *p*_*j*−1_/*u*_*j*−1_**end***u*_0_*_′_* = 5*i_1_*R + *u_1_***while** (*u*_0_*_′_*! = *u*_0_)


The reverse algorithm is a specific algorithm for Equations (2) and (3), which is able to reach the same solutions as Equations (9). The Newton iteration algorithm is a common algorithm for nonlinear equations. As shown in Figure 6 and Algorithm 1, the solution procedure of reverse algorithm is simpler than Newton iteration algorithm. When *N* (*N* = number of nodes) equals 150, the calculating time of reverse algorithm is about one or two minutes utilizing matlab. The calculating time of Newton iteration is about two or three minutes under the same condition.

## 4. Simulation and Verification of the Model 

We elaborate on the verification of the accuracy of the Newton iteration algorithm and reverse algorithm in Section 4.1. In the laboratory, we perform a test to verify the model. The experimental results verify the accuracy of the model, which is detailed in Section 4.2. In Section 1, it is mentioned that the length of streamer (HQI-Seis) can reach only 7 km with a tracking spacing of 3.125 m. In Section 4.3, we discuss doubts in detail. In further study about the model, we find the reason why MSESS can only support a finite number of nodes under given system parameters.

### 4.1. Verification of the Accuracy of Two Algorithms

**Figure 7:**
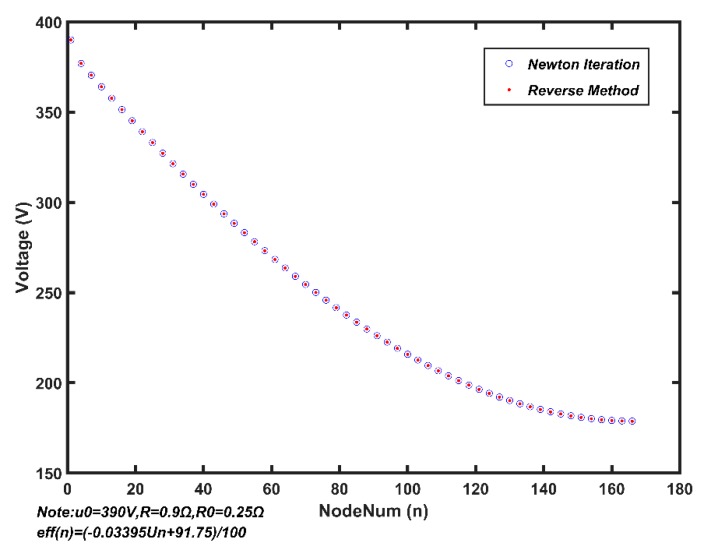
Calculation results of Newton iteration and reverse algorithms.

We use the Newton iteration algorithm and reverse algorithm to solve Equations (9) to find the maximum number of nodes that MSESS can operate normally under measured parameters. The results show that the equations have no solution when *N* increases to 166. The calculating result of the Newton iteration algorithm does not converge, while the reverse algorithm falls into an infinite loop. Neither algorithm produces a result. The calculated result shows that the maximum number of nodes under the current parameters is 165. The voltage of each node is shown in Figure 7. The blue circles represent the result of the Newton iteration algorithm and the red dots stand for the result of the reverse algorithm. The results of the Newton iteration algorithm and the reverse algorithm are nearly the same, which verifies the accuracy of the two algorithms.

### 4.2. Verification of the Model—Test in Laboratory

To verify the model under laboratory conditions, we set up a test platform of 1300 m (*N* = 13). The laboratory’s high-voltage DC power supply is used to simulate the power supply of the geophysical survey vessel and the electronic load is used to simulate DAS. The resistance of the power cable (100 m) was about 2.06 Ω. 

**Figure 8:**
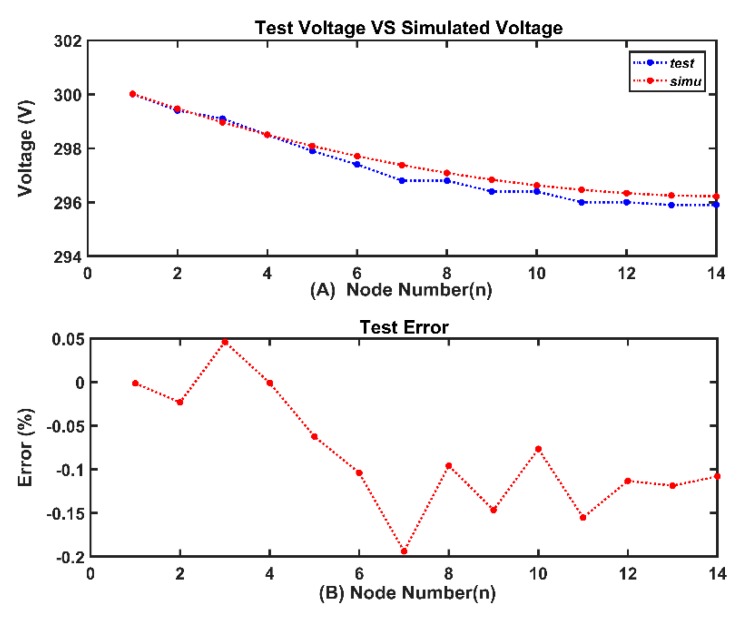
Test result: (**A**) comparison of simulated voltage and test voltage (**B**) error percentage of voltage.

Figure 8 illustrates the comparison of test results and simulated results. The maximum error of voltage was less than 0.2%. This error comes from the inaccuracy of the electronic load. Considering the minor error and the source of the error, the experiment proves that the model is accurate.

### 4.3. Reason for the Existence of Maximum Number of Nodes

**Figure 9:**
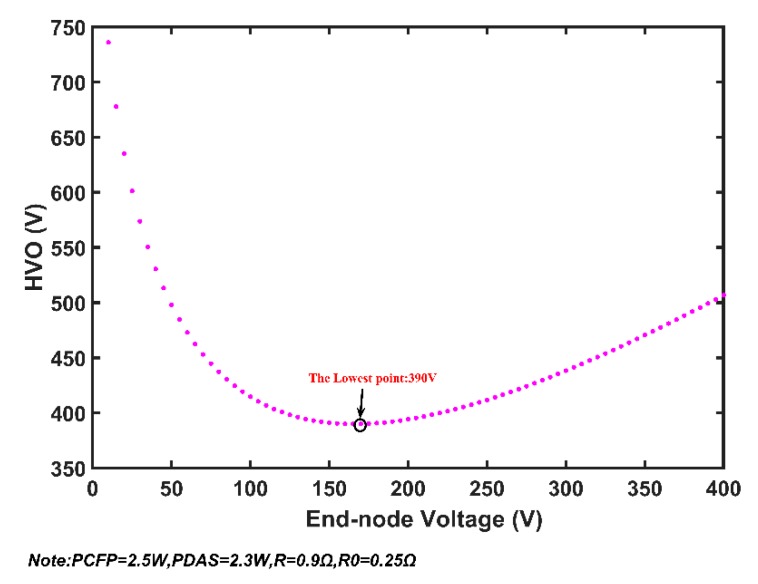
Relationship of high-voltage direct current output (HVO) and end-node voltage.

**Figure 10:**
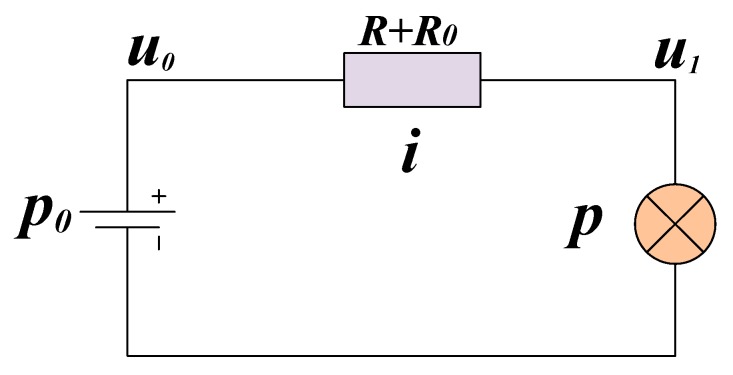
Simplified model of one node.

Assuming that the model and algorithms are accurate, the results of the two algorithms indicate that MSESS can operate normally at the measured parameters only when the number of nodes is less than or equals 165. If one more node is added, the equations will have no solution and the system will misbehave. There should be some reason for this phenomenon. We undertook further research to determine a reasonable explanation. The relationship between HVO (u_0_ in Figure 4) and end-node voltage (u_n_ in Figure 4) is presented in Figure 9 when *N* equals 165. The figure illustrates the following problem: if HVO exceeds the value of the lowest point (390 V), there will be two end-node voltage values, which is inconsistent with the actual situation. When the system is stable, the end-node voltage should be a unique value. Using one node (*N* = 1) as an example, we conduct analyses using the simplified system structure illustrated in Figure 10.

The equations for finding the values of *u*_1_ and *i* is shown as simultaneous Equations (10):

(10) $$\{\begin{array}{c} u_{0}=u_{1}+i\left(R+R_{0}\right)\\ p=u_{1}i \end{array}$$

**Figure 11:**
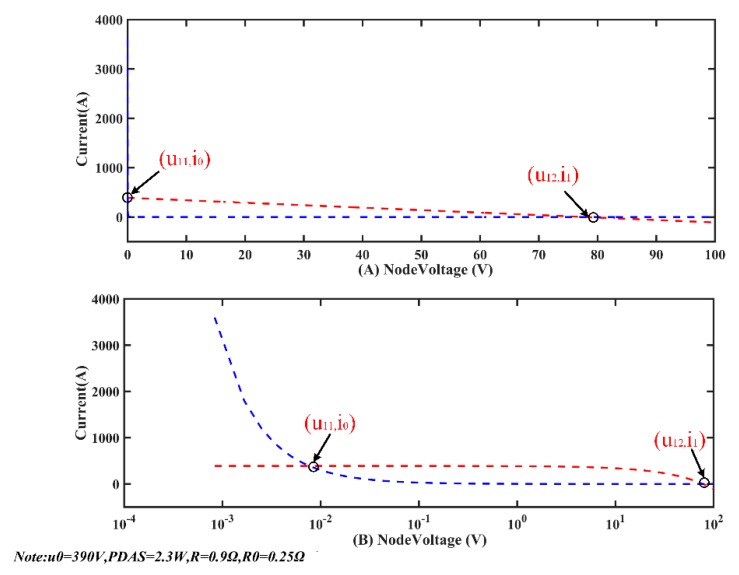
Solution result of one node.

The system parameters are *u*_0_ = 390 V, *R* = 0.9 Ω, *R*_0_ = 0.25 Ω, and *p* = 2.3/eff (1). The result of the calculations, in both a linear coordinate system (on the top) and a logarithmic coordinate system (at the bottom) is shown in Figure 11. The red curve represents the relationship between *u*_1_ and *i* shown in the first equation of (10), and the blue curve represents the relationship between *u*_1_ and *i* in the second equation in (10). The intersection of the two curves are the solutions of Equations (10). From the curve on the logarithmic coordinate axes, it can be seen clearly that the equations have two mathematical solutions: (*u*_11_, *i*_0_) and (*u*_12_, *i*_1_). The solution (*u*_11_, *i*_0_) expresses as *u*_11_ is small (10^−2^), while *i*_0_ is very large (10^2^). The characteristics of (*u*_12_, *i*_1_) are opposite. (*u*_11_, *i*_0_) and (*u*_12_, *i*_1_) are named as math solution. When MSESS runs, there is only one solution corresponding to the actual situation. The current cannot be hundreds of ampere in a functioning system. Therefore, (*u*_12_, *i*_1_) corresponds to the actual parameter when the system is running. (*u*_12_, *i*_1_) is named as the physical solution.

**Figure 12:**
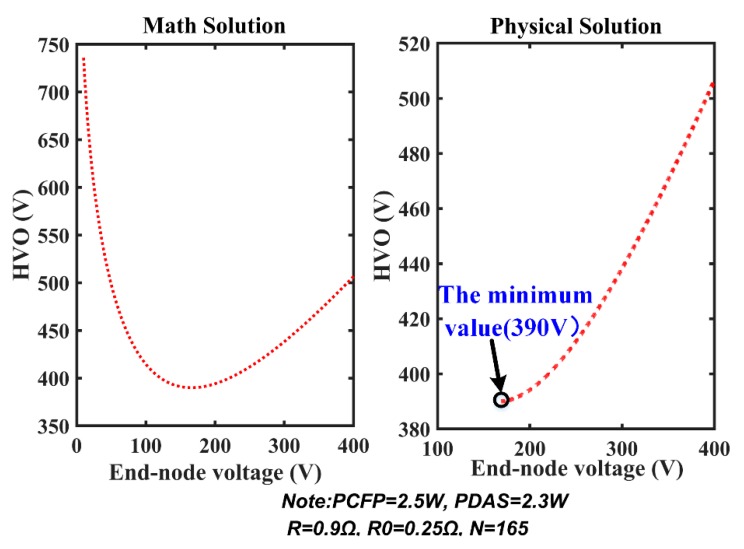
Comparison of the mathematical solution and physical solution.

Our analysis of the special case (*N* = 1) indicates the existence of mathematical solution and physical solution. When *N* increases to 165, the relationship of the end-node voltage and HVO is shown in Figure 12. In the left part, once HVO exceeds the lowest point, the end-node voltage will have two numerical values. The two solutions correspond to the mathematical solution. The right part corresponds to the physical solution, which is meaningful for actual situation. The lowest point of the curve represents the minimum value of HVO at which MSESS will operate normally at 165 nodes.

**Figure 13:**
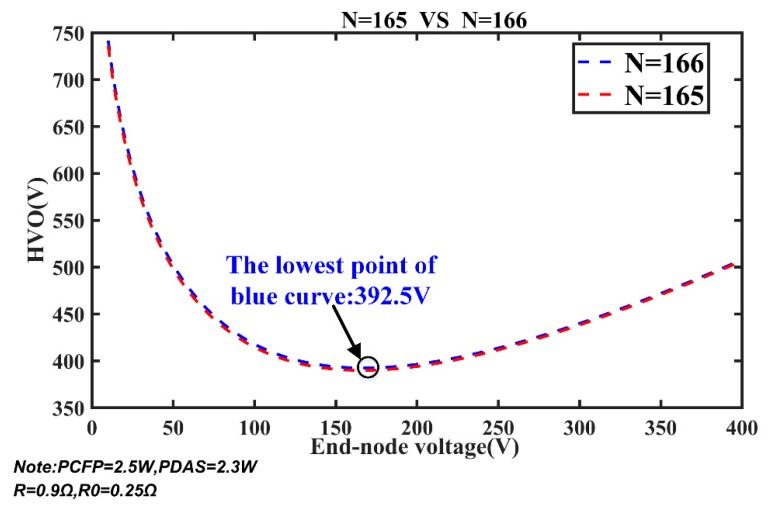
Relationship of HVO and end-node voltage as *N* = 165 and *N* = 166.

Further verification is illustrated in Figure 13. The blue curve shows the relationship of HVO and the end-node voltage when *N* equals 166. The lowest point of blue curve is 392.5, which is the minimum value of HVO to make MSESS work normally. Once the value of HVO is less than 392.5 V, MSESS is unable to function when *N* equals 166. The red curve represents the relationship between HVO and end-node voltage when *N* equals 165. The lowest point of red curve is 390. It is obvious that HVO must be at least 392.5 V to make MSESS operate normally at 166 nodes, and there is no solution for the equations if HVO is 390 V. If HVO is set to 390 V and *N* equals 166, what will happen in actual situation?

**Figure 14:**
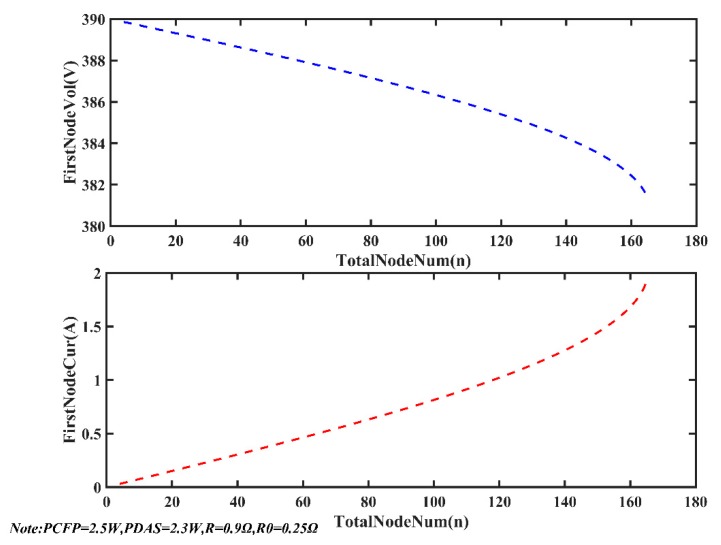
First-node voltage and current vary as the total number of nodes increases.

Figure 14 shows the varying curve of the first-node current (i_1_ in Figure 4) and voltage (u_1_ in Figure 4) as the total number of nodes (here it stands for *N*, the maximum value equals 165) increases. When system parameters (u_0_, R, R_0_, PCFP, PDAS) are constant, the first-node current increases sharply and the first-node voltage drops sharply as the total number of nodes increases to 165. From the trend of the curve, the current of the first node is an infinite value when *N* equals 166. Therefore in an actual situation, if HVO is set to 390 V and *N* equals 166, the output current of the power supply will be an infinite value. The power supply will turn on the overcurrent protection and disconnect the output. 

From the analysis above, it can be concluded that for given system parameters and a certain number of nodes, HVO must have a minimum value to make the system work properly. From another perspective, once the system parameters and HVO are specified, the number of nodes with which the system works properly is determined. When the number of nodes exceeds the maximum value, the system current will be an infinite value and the power supply will shut down due to the function of overcurrent protection. This is why the number of nodes supported by “HQI-Seis” is limited. 

## 5. Discussion of the Model 

According to the model, the line loss on the power cable and the power consumption of each node (*p*_1_~*p*_n_ in Figure 4) affect the length of MSESS. We have a detailed analysis in Section 5.1. To calculate the maximum length of MSESS under given system parameters quickly, we develop a kind of software. In Section 5.2, the function of the software is introduced.

### 5.1. Factors Affecting the Length of the Streamer

**Figure 15:**
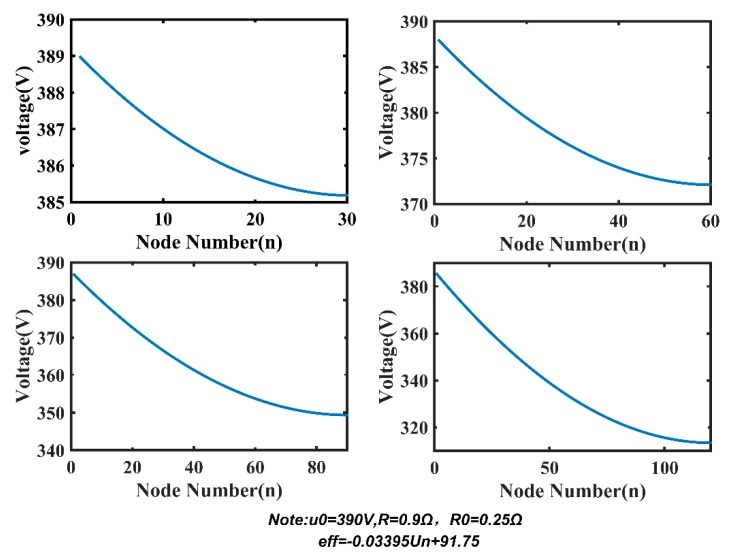
Voltage distribution.

**Figure 16:**
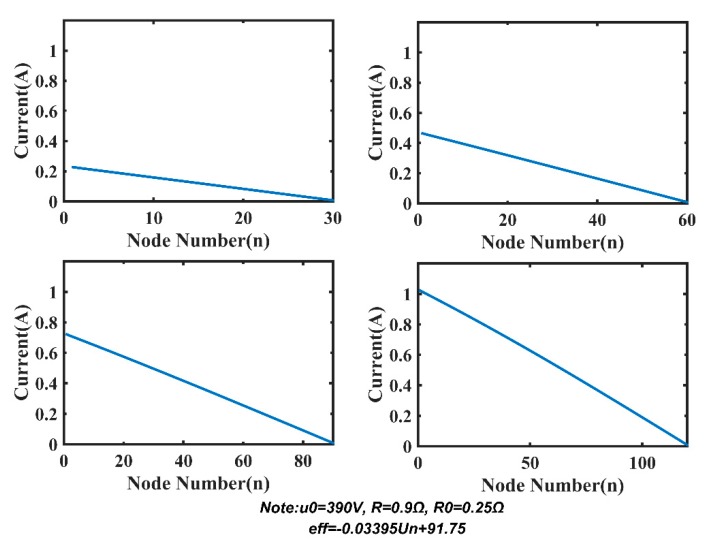
Current distribution.

Based on the optimized model, we research two factors affecting the length and power consumption of MSESS, including R and PDAS. Figure 15 demonstrates voltage distribution with 30, 60, 90, and 120 nodes under the condition that system parameters are u_0_ = 390 V, R = 0.9 Ω, R_0_ = 0.25 Ω, and PDAS = 2.3 W. We can see that the voltage on the power line remains relatively stable when there are only 30 or 60 nodes. When the number of nodes increases to 90 or 120, the voltage drops rapidly due to line loss on the power line. Corresponding to Figure 15, the current distribution is shown in Figure 16. As the number of nodes increases, the total current increases accordingly, which results in a rise in system power consumption.

**Figure 17:**
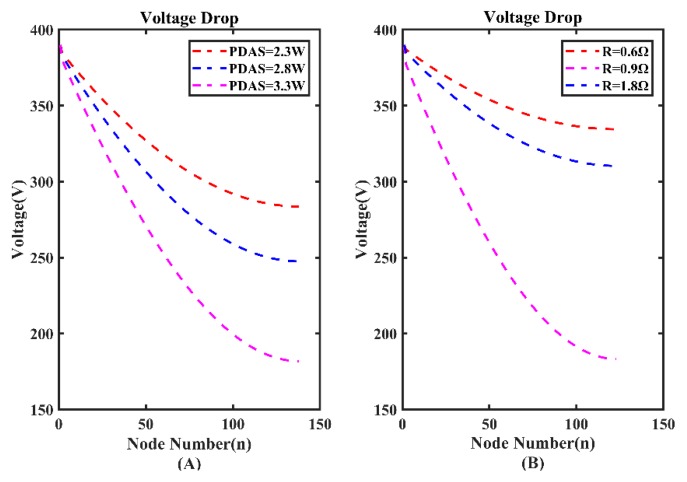
(**A**) Voltage drop with different power consumption of data acquisition station (PDAS). (**B**) Voltage drop with different R.

The value of power consumption of each node (*p*_1_~*p*_n_ in Figure 4) affects the length of MSESS. Figure 17A illustrates the curve of voltage drop with 137 nodes. It is obvious that voltage descends rapidly when PDAS increases from 2.3 W to 3.3 W. For further research, we calculate the maximum number of nodes using a range of PDAS (Table 1). As PDAS changes from 3.3 W to 2.3 W, the maximum number of nodes increases from 137 to 165. Therefore, reducing the power of a single node is an effective way to raise the number of nodes supported by MSESS.

Another important factor affecting the length of MSESS is the value of R, which is the key to line loss. In the laboratory, the power supply line is composed of two 14 AWG (American wire gauge) lines (R = 0.9 Ω). We run a simulation under the condition that R is 0.6 Ω (the value of R of three 14 AWG lines) and 1.8 Ω (the value of R of one 14 AWG line). The results of the simulation are shown in Figure 17B. When the value of R increases, the voltage drops rapidly. The maximum number of nodes with different value of R is shown in Table 2.

Reducing the value of R is an effective approach to increase the length of MSESS. Due to the harsh working environment of marine seismic exploration, it is impossible to reduce the impedance by using multi-strand power lines in parallel in engineering applications. This shows reducing the value of PDAS is a more effective way to design long MSESS.

### 5.2. Predicting the Maximum Length of MSESS

**Figure 18:**
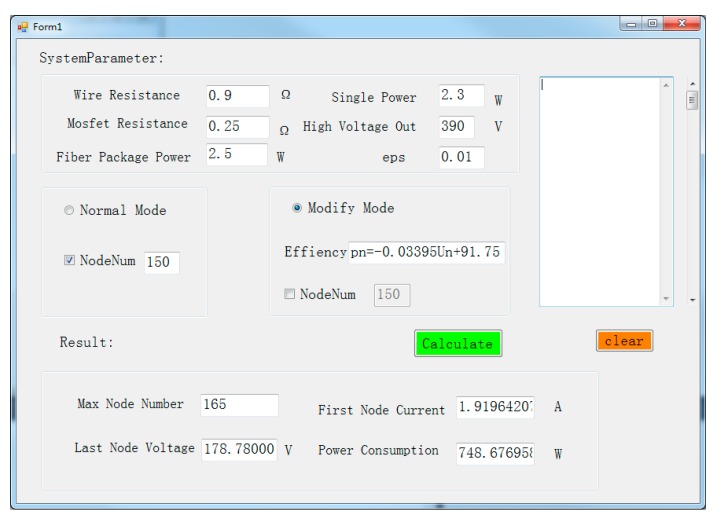
Software interface.

The length of the MSESS affects the depth of the exploration target layer, which is critical in designing the system. In order to quickly acquire the maximum length that can be supported by MSESS under certain parameters, we use C# to develop a human-computer interaction interface program based on the reverse algorithm. The graphical interface is shown in Figure 18. There are two main functions of the software. One function is to find the maximum number of nodes supported by MSESS under given system parameters. The other is to obtain other parameters of the system (end-node voltage, system power consumption, etc.) given known number of nodes. When system parameters (such as supply voltage, single node power consumption, wire resistance, etc.) are given, the maximum number of nodes can be calculated quickly to predict the maximum length supported by MSESS.

## 6. Conclusions

This paper researches the power system used in marine seismic exploration. A model to analyze the voltage distribution and current distribution is built. Non-linear equations are established for the model, which are solved by the Newton iteration algorithm and reverse algorithm. A test platform of 1300 m is built in the laboratory and the accuracy of the model is verified based on the analysis of measured results and simulated results. With further research, we discover why “HQI-Seis” can reach only 7 km under given system parameters. We have a detailed discussion about the two factors (the value of R and PDAS) affecting the length and power consumption of MSESS. By research, reducing the value of PDAS is a more effective way to design long MSESS. Based on the reverse algorithm, we develop a kind of software that is able to quickly calculate the maximum number of nodes that the system can support. System designers can predict the maximum length of MSESS conveniently using the software. The research results of this paper are of great significance to design long MSESS. The research methods in this paper could also be applied to relevant works such as large-scale sensor networks in land seismic exploration.

## Figures and Tables

**Table 1:** Maximum number of nodes under different PDAS.

PDAS (W)	HVO (V)	End-Node Voltage (V)	*p*_0_ (W)	Maximum Number of Nodes
2.3	390	178.77	748.677	165
2.8	390	181.99	814.9769	149
3.3	390	181.79	883.6328	137

**Table 2:** Maximum number of nodes with different R.

R (Ω)	HVO (V)	End-Node Voltage (V)	*p*_0_ (W)	Maximum Number of Nodes
0.6	390	176.97	879.04	193
0.9	390	178.77	748.677	165
1.8	390	183.18	548.28	122
